# Supplementary material for: Risk factors for sacrococcygeal pilonidal sinus: a systematic review and meta-analysis supplemented by genetic causal assessment
Source: Front Surg. 2026 Jan 7;12:1718589. doi: 10.3389/fsurg.2025.1718589 (PMC12819706; doi:10.3389/fsurg.2025.1718589)
Supplement: Supplementary file 2 [file Datasheet2.zip › Supplementary Data 2/MR_pipeline_p5e-6/leaveone_plots/ukb-ppp-COL3A1-OID30379.finngen_R12_L12_PILONIDALCYST.pdf]

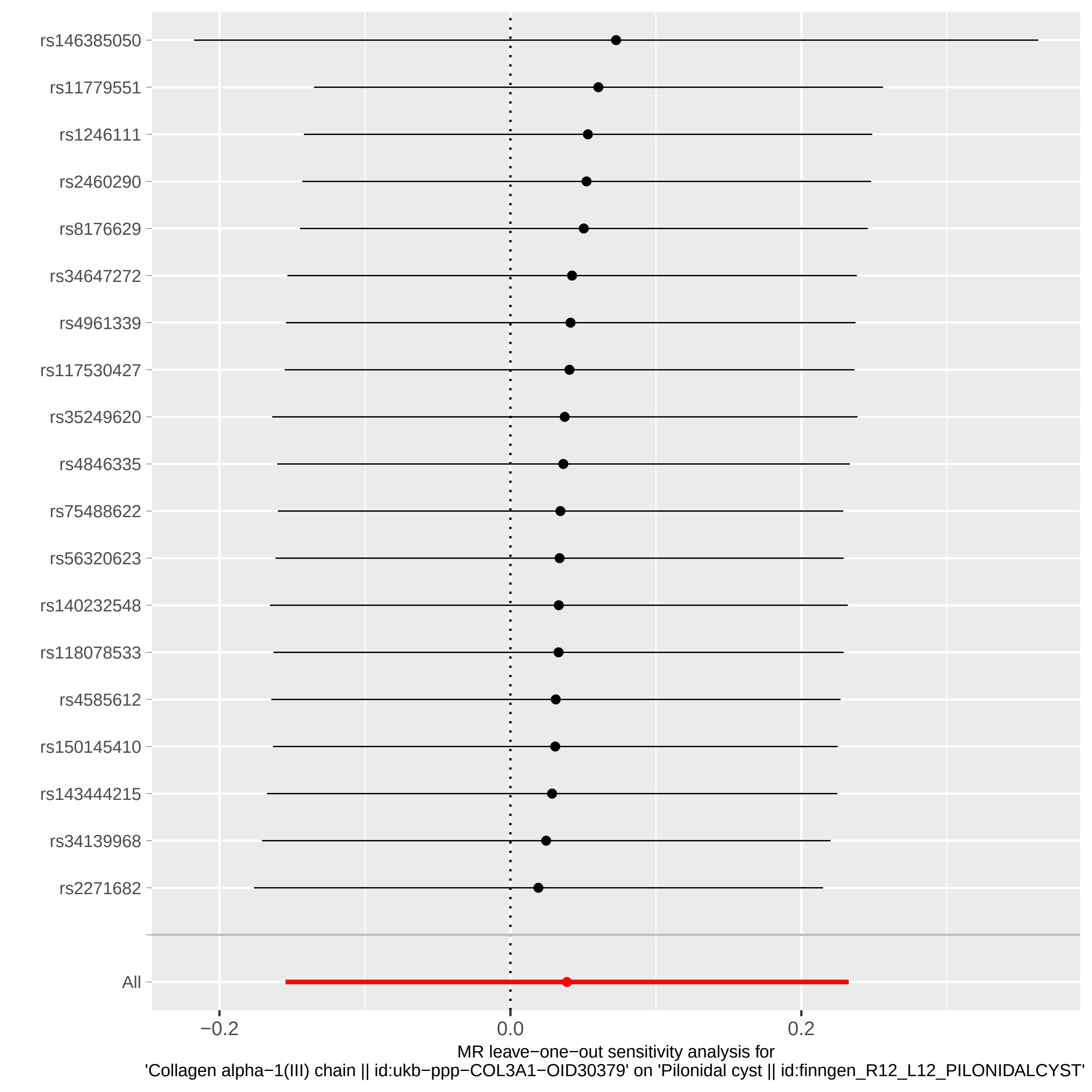
rs146385050
rs11779551
rs1246111
rs2460290
rs8176629
rs34647272
rs4961339
rs117530427
rs35249620
rs4846335
rs75488622
rs56320623
rs140232548
rs118078533
rs4585612
rs150145410
rs143444215
rs34139968
rs2271682
All
−0.2
0.0
0.2
MR leave−one−out sensitivity analysis for
'Collagen alpha−1(III) chain || id:ukb−ppp−COL3A1−OID30379' on 'Pilonidal cyst || id:finngen_R12_L12_PILONIDALCYST'
